# Subgingival Microbiome and Specialized Pro-Resolving Lipid Mediator Pathway Profiles Are Correlated in Periodontal Inflammation

**DOI:** 10.3389/fimmu.2021.691216

**Published:** 2021-06-10

**Authors:** Chun-Teh Lee, Ruoxing Li, Lisha Zhu, Gena D. Tribble, W. Jim Zheng, Brittney Ferguson, Krishna Rao Maddipati, Nikola Angelov, Thomas E. Van Dyke

**Affiliations:** ^1^ Department of Periodontics and Dental Hygiene, School of Dentistry, The University of Texas Health Science Center at Houston, Houston, TX, United States; ^2^ School of Biomedical Informatics, The University of Texas Health Science Center at Houston, Houston, TX, United States; ^3^ Department of Pathology, Wayne State University, Detroit, MI, United States; ^4^ Center for Clinical and Translational Research, The Forsyth Institute, Cambridge, MA, United States; ^5^ Department of Oral Medicine, Infection, and Immunity, Faculty of Medicine, Harvard University, Boston, MA, United States

**Keywords:** computational biology, host microbial interaction, inflammation, metabolomics, microbiota, lipidomics, periodontitis

## Abstract

Failure of resolution pathways in periodontitis is reflected in levels of specialized pro-resolving lipid mediators (SPMs) and SPM pathway markers but their relationship with the subgingival microbiome is unclear. This study aimed to analyze and integrate lipid mediator level, SPM receptor gene expression and subgingival microbiome data in subjects with periodontitis vs. healthy controls. The study included 13 periodontally healthy and 15 periodontitis subjects that were evaluated prior to or after non-surgical periodontal therapy. Samples of gingival tissue and subgingival plaque were collected prior to and 8 weeks after non-surgical treatment; only once in the healthy group. Metabololipidomic analysis was performed to measure levels of SPMs and other relevant lipid mediators in gingiva. qRT-PCR assessed relative gene expression (2^-ΔΔCT^) of known SPM receptors. 16S rRNA sequencing evaluated the relative abundance of bacterial species in subgingival plaque. Correlations between lipid mediator levels, receptor gene expression and bacterial abundance were analyzed using the Data Integration Analysis for Biomarker discovery using Latent cOmponents (DIABLO) and Sparse Partial Least Squares (SPLS) methods. Profiles of lipid mediators, receptor genes and the subgingival microbiome were distinct in the three groups. The strongest correlation existed between lipid mediator profile and subgingival microbiome profile. Multiple lipid mediators and bacterial species were highly correlated (correlation coefficient ≥0.6) in different periodontal conditions. Comparing individual correlated lipid mediators and bacterial species in periodontitis before treatment to healthy controls revealed that one bacterial species, *Corynebacterium durum*, and five lipid mediators, 5(S)6(R)-DiHETE, 15(S)-HEPE, 7-HDHA, 13-HDHA and 14-HDHA, were identified in both conditions. Comparing individual correlated lipid mediators and bacterial species in periodontitis before treatment to after treatment revealed that one bacterial species, *Anaeroglobus geminatus*, and four lipid mediators, 5(S)12(S)-DiHETE, RvD1, Maresin 1 and LTB4, were identified in both conditions. Four *Selenomonas* species were highly correlated with RvD1, RvE3, 5(S)12(S)-DiHETE and proinflammatory mediators in the periodontitis after treatment group. Profiles of lipid mediators, receptor gene and subgingival microbiome are associated with periodontal inflammation and correlated with each other, suggesting inflammation mediated by lipid mediators influences microbial composition in periodontitis. The role of correlated individual lipid mediators and bacterial species in periodontal inflammation have to be further studied.

## Introduction

In the United States, around 60 million adults over 30 years of age have periodontitis (42.2%) with 7.8% having severe periodontitis (1). Periodontitis is a biofilm induced chronic inflammatory disease characterized by gingival inflammation and destruction of alveolar bone. Disproportionate host responses and microbiota dysbiosis are the two major etiological factors (2). Periodontal tissue damage is primarily mediated by bacterially induced immune responses. The composition of the subgingival microbiota is associated with the inflammatory status in periodontal tissue (3). It is hypothesized that the change of local environment induced by inflammation results in the shifts of the subgingival microbiota (4, 5). Many studies have shown that the composition of the subgingival microbiota shifts following periodontal therapy performed to control inflammation (6, 7). In periodontitis, the inflamed tissues with deep periodontal pocket provide an anaerobic environment with breakdown products of tissue destruction, plasma proteins and hemoglobin as nutrients for the growth of several anaerobic gram-negative bacteria, resulting in microbiota shifts. Once inflammation is controlled, the environment is not suitable for periodontal pathogens anymore and commensal microbiota can be re-established in a homeostatic relationship with the host.

Resolution of inflammation is a proactive process induced by specialized pro-resolving lipid mediators (SPMs), including lipoxins, resolvins, protectins and maresins, that bind to specific G protein-coupled receptors on a variety of cells. In the resolution phase of inflammation, there is decreased infiltration of neutrophils, reduced levels of pro-inflammatory cytokines and lipid mediators, and increased recruitment of resolving macrophages that clear the lesion by efferocytosis (8, 9). It has been demonstrated that SPMs control inflammatory diseases, such as inflammatory bowel disease (10), diabetes (11) and periodontitis (12) in the preclinical models. Specifically, in experimental periodontitis, SPMs, such as resolvin E1, topically applied on gingiva can prevent bone loss, regenerate the lost bone, change gene expression patterns in gingiva and result in shifts of the oral microbiota (13, 14). Resolution of inflammation induced by SPMs can influence the composition of the subgingival microbiota in periodontal inflammation.

In humans, SPMs have been found in milk (15), serum, lymphoid tissue (16), saliva and gingival crevicular fluid (17, 18). The levels of SPMs and related lipid mediators in various specimens are associated with the inflammatory status of mammary glands (15), the stability of atherosclerotic plaques (19), the severity of tuberculous meningitis (20) and the disease status of periodontitis (17, 18). Recently, SPMs, SPM pathway markers and SPM corresponding receptor genes are identified in gingival tissues, the periodontal inflammation site (21). Profiles of these lipid mediators and receptor genes are associated with the severity of inflammation. However, the associations between these lipid mediators and the oral microbiome have not been explored. Since preclinical studies demonstrated that resolution of inflammation induced by SPMs is associated with shifts in the taxonomic composition of the oral microbiota (13, 14), there is a need to investigate the clinical relationship between profiles of SPM relevant lipid mediators and subgingival microbiome in periodontal inflammation to clarify how inflammatory reactions mediated by SPMs affect subgingival microbial composition in humans and vice versa.

The aim of this study was to assess correlations between levels of lipid mediators including SPMs and SPM pathway markers, expression of SPM receptor genes in human gingiva and the relative abundance of bacterial species in subgingival plaque. The results of this study indicate the potential interactions between lipid mediators and bacterial species in different periodontal inflammatory conditions.

## Materials and Methods

### Clinical Study Design

The study was conducted in accordance with the guidelines of the World Medical Association’s Declaration of Helsinki and approved by the University of Texas Health Science Center at Houston (UTHealth) Committee for the Protection of Human Subjects (HSC-DB-16-0167). All participants provided written informed consent. The inclusion criteria were subjects aged ≥18 with ≥24 teeth and no history of systematic periodontal therapy within the past two years. These subjects should not have received anti-inflammatory drugs for more than one week and systemic antibiotics within three months before sample collection, did not routinely take fish oil supplements, had no presence of diabetes mellitus or any systemic condition that entails a diagnosis of “systemic disorders that have a major impact on the loss of periodontal tissues by influencing periodontal inflammation” (22), were not pregnant, and were not current users of tobacco products or nicotine replacement medication.

A comprehensive periodontal charting and examination were performed for all subjects. Probing depth (PD), level of free gingival margin (FGM), clinical attachment level (CAL) and presence of bleeding of probing (BOP) were measured at six sites per tooth. Full mouth series (FMS) radiographs were obtained prior to all treatments for all subjects to confirm alveolar bone level. The subjects in the healthy group were required to meet all inclusion criteria, as well as have all teeth with a probing depth of ≤3mm, clinical attachment loss of ≤2mm (except teeth with mid-buccal or lingual gingival recession), and radiographic bone levels ≤2mm from the cementoenamel junction (CEJ) (no radiographic bone loss due to periodontitis). The subjects in the periodontitis group were required to present with ≥8 teeth with a probing depth of ≥5mm, clinical attachment loss of ≥3mm, and radiographic bone levels >2mm from the CEJ.

### Clinical Sample Collection

Subjects in the healthy group were seen for one clinical research visit, prior to prophylaxis. During the visit, four gingival biopsy samples were obtained from interproximal sites (mesiobuccal, distobuccal, mesiolingual, or distolingual sites of each tooth) of two representative posterior teeth and subgingival plaque samples were obtained from eight interproximal sites of the same two teeth. Subjects in the periodontitis group were seen for two clinical visits; the first visit being prior to the first scaling and root planing (SRP) appointment, and the second visit eight weeks following completion of the second SRP appointment. In these two visits, gingival biopsies and subgingival plaque samples were collected from interproximal sites of the same two representative posterior teeth. The most severely affected teeth with more bone loss than other teeth were selected for gingival biopsy and plaque sample collection. All first-visit samples collected in the periodontitis subjects were from sites with deep probing depths (≥ 5mm).

Gingival biopsies were performed as previously described (21). At each site for gingival sample collection, intrasulcular incisions were made from the papilla zenith toward the base of the interproximal papillae, but not to exceed the nearest line angle of each tooth. A subsequent horizontal incision was made at the base of the interproximal papillae to connect the intrasulcular incisions. The incisions extended to the alveolar bone, and split thickness papillae were partially elevated to permit the harvesting of the interproximal gingival tissue. The gingival sample was required to include epithelium and the underlying supracrestal connective tissue. Of the four gingival tissue samples collected, the sample selected for Lipid Mediator-SPM metabololipidomic analysis was from the site with the deepest probing depth and the sample selected for real-time quantitative reverse transcription assay (qRT-PCR) was from the site with the second-deepest probing depth. The other two gingival tissue samples were stored for future use.

Prior to subgingival plaque collection, supragingival plaque was removed with a separate sterilized scaler. Each subgingival plaque sample was collected using a sterile end of a Gracey mini-curette (Hu-Friedy, Chicago, IL, USA), which was inserted into the depth of the sulcus, with a standardized 20 single strokes. The plaque sample collected from one single site was placed in a microcentrifuge tube with 150µl of Tris-EDTA (TE) buffer, using an up and down motion to ensure deposition of the plaque sample into the TE buffer. The tube was immediately placed in liquid nitrogen and then transferred and stored at -80°C. Only one of the eight plaque samples was processed for 16S rRNA sequencing where possible to avoid affecting microbiome data by pooling samples from multiple sites with different periodontal conditions. Eight plaque samples were collected because sometimes the DNA concentration of a single site could be low. For one subject, the DNA concentration of the single site was too low for PCR amplification, thus samples from two sites were combined. Generally, the selected plaque samples were from sites with the deepest probing depth matched to the sites for Lipid Mediator-SPM metabololipidomics when possible.

### Lipid Mediator-SPM Metabololipidomics for Gingival Tissue Samples

The gingival tissue samples were quantitatively analyzed for levels of SPMs, SPM pathway markers and proinflammatory mediators using Lipid Mediator-SPM metabololipidomics as described earlier (21, 23). The quantities of these lipid mediators in gingival tissue samples are presented as mean ± standard deviation ng per mg of the total protein in each sample. The lower limit of quantitation is 0.015ng.

### Expression of SPM Receptor Genes in Gingival Tissue Samples *via* qRT-PCR

Expression of SPM corresponding receptor genes was analyzed with qRT-PCR, utilizing the TaqMan Gene Expression Assays Protocol (Applied Biosystems, Foster City, CA, USA). Details of the experiment were previously described (21). The TaqMan Assay primers included *GAPDH* (assay ID: Hs99999905_m1, Applied Biosystems) as the housekeeping gene compared to *ALX (FPR2)* (assay ID: Hs00265954_m1, Applied Biosystems), *BLT1 (LTB4R)* (assay ID: Hs00175124_m1, Applied Biosystems), *ChemR23 (ERV1)* (assay ID: Hs01386063_m1, Applied Biosystems)*, GPR18 (DRV2)* (assay ID: Hs00245542_m1, Applied Biosystems)*, GPR32 (DRV1)* (assay ID: Hs00265986_s1, Applied Biosystems)*, GPR37* (assay ID: Hs0017374_m1, Applied Biosystems),and *LGR6* (assay ID: Hs00663887_m1, Applied Biosystems), which were labeled with FAM dye. The calculated cycle threshold (Ct) values from each sample were obtained and relative expression (2^-ΔΔCt^) was calculated to determine expression of specific SPM receptor genes between groups.

### 16S rRNA Sequencing for Subgingival Plaque Samples

DNA extraction of one representative plaque sample was performed utilizing the protocol of a MO BIO PowerSoil DNA Isolation Kit (MO BIO Laboratories, Carlsbad, CA USA) (24). Eukaryotic and prokaryotic cells from the plaque sample were lysed with garnet beads to release DNA. The DNA was isolated on a PowerSoil spin filter and subsequently washed and collected as an eluate in a separate collection tube. The DNA eluate was measured on the UV-Vis spectrophotometer (Nanodrop, Thermo-Fisher Scientific, Waltham, MA, USA) to determine the amount of DNA in the sample. Then DNA samples were processed for 16S rRNA Sequencing (LC Biosciences, Houston, TX). The 16S rRNA V3-V4 regions were amplified *via* PCR and sequenced on the Illumina MiSeq platform (Illumina Inc., San Diego, CA, USA). The amplification primers (forward primer (338F): 5’-ACTCCTACGGGAGGCAGCAG-3’; reverse primer (806R): 5’-GGACTACHVGGGTWTCTAAT-3’) contained adapters for MiSeq sequencing and single-index barcodes that resulted in PCR products that were pooled and sequenced directly. Read pairs were de-multiplexed based on barcodes and merged. All of the samples were then processed and sequenced together, and the cleaned, merged data was imported into CLC Genomics Workbench with the Microbial Genomics module. The 16S rRNA gene sequences were allocated to specific operational taxonomic units (OTUs) at 98% identity using the Human Oral Microbiome Database (HOMD). OTUs without a match to the HOMD database were given an OTU number, and genus/species were identified by BLAST search against the bacterial 16S rRNA database (25).

### Statistical Analysis

The Lipid Mediator-SPM metabololipidomics data and qRT-PCR data were first log transformed, and Wilcoxon rank sum test was used to detect differentially expressed lipid mediators and receptor genes between two conditions except for comparison between the periodontitis prior to SRP group and the periodontitis after SRP group, where Wilcoxon signed rank test was applied. The p-values were adjusted using the False Discovery Rate (FDR) multi-test correction method. Data were considered statistically significant when p<0.05 (21).

For the subgingival microbiome, community diversity (alpha and beta diversity) was assessed using the Microbial Genomics Diversity module of CLC Genomics Workbench. OTUs from the abundance table were aligned using MUSCLE with a required minimum abundance of 100. Aligned OTUs were used to construct a phylogenetic tree using Maximum Likelihood Phylogeny using the Neighbor Joining method and the Jukes Cantor substitution model. Rarefication analysis was done by sub-sampling the OTU abundances in the different samples at a range of depths from 1 to 100,000; the number of different depths sampled was 20, with 100 replicates at each depth. The OTUs with relative abundance less than 0.01% were excluded. Alpha diversity measures were calculated for observed OTUs and Shannon index. Statistical significance in alpha diversity between groups was calculated with Student’s t-test or paired t-test. PERMANOVA (Permutational Multivariate Analysis of Variance) analysis was used to detect significant differences in Beta diversity between groups, and comparisons were visualized using Principal Coordinate Analysis (PCoA). Diversity measures were calculated using the Bray-Curtis formula.

Differences in taxa abundance of subgingival plaque samples between the three groups were identified using DESeq2 (26). OTUs were selected through DESeq2 testing. The OTUs were color-coded according to the phyla they belong to and plotted according to their log2 fold change. The relative fold change was calculated between the three subject groups.

R random Forest package (27) was used to calculate the relative abundance of OTUs. Those OTUs with mean decrease Gini larger than 0.15 were selected for subsequent integration analysis. The lipid mediator levels, SPM receptor gene expression levels and bacterial abundance were first normalized to zero mean and unit variance, and then integrated and analyzed using Data Integration Analysis for Biomarker discovery using a Latent cOmponents (DIABLO) method implemented in R mixOmics package (28). The resulting profile correlations were visualized in DIABLO. Correlations between individual lipid mediators and bacterial species in each subject group were further analyzed using the sparse partial least squares (SPLS) method (29) implemented in the R mixOmics package. The data at the genus level were also analyzed using the same methods.

## Results

### Composition of the Subgingival Microbiome

Thirteen periodontally healthy subjects and 15 periodontitis subjects before and after treatment were included in this study. All periodontitis subjects were diagnosed with generalized/localized periodontitis, Stage II or III, Grade B or C (**Supplementary Table 1**). In periodontitis subjects, the mean probing depth of sample collection sites was significantly reduced following non-surgical periodontal therapy (SRP); however, the average PD after treatment remained significantly greater than in health (**Table 1**).

**Table 1:** Characteristics of subjects and sample collection sites.

	Healthy (H)	Periodontitis before non-surgical therapy (P)	Periodontitis after non-surgical therapy (A)	p-value (H versus P/A)	p-value (P versus A)
**Age**	39.31 ± 15.80	51.20 ± 9.99	51.20 ± 9.99	0.02/0.02	NA
**Gender (Male/Female)**	6/7	8/7	8/7	0.71/0.71	NA
**PD (mm) of sites for metabololipidomics**	2.85 ± 0.38	6.47 ± 1.13	5.33 ± 1.76	<0.01/<0.01	0.01
**PD (mm) of sites for qRT-PCR**	2.85 ± 0.38	5.87 ± 1.13	4.13 ± 1.51	<0.01/0.01	<0.01
**PD (mm) of sites for 16S RNA sequencing**	2.85 ± 0.38	6.47 ± 1.13	5.27 ± 1.69	<0.01/<0.01	<0.01
**CAL (mm) of sites for metabololipidomics**	0.31 ± 0.48	6.00 ± 1.73	5.33 ± 2.19	<0.01/<0.01	0.06
**CAL (mm) of sites for qRT-PCR**	0.38 ± 0.51	5.33 ± 1.63	4.60 ± 1.76	<0.01/<0.01	0.01
**CAL (mm) of sites for 16S RNA sequencing**	0.31 ± 0.48	6.00 ± 1.73	5.27 ± 2.19	<0.01/<0.01	0.03
**FGM (mm) of sites for metabololipidomics**	2.54 ± 0.52	0.47 ± 1.60	0.00 ± 1.89	<0.01/<0.01	0.29
**FGM (mm) of sites for qRT-PCR**	2.46 ± 0.52	0.53 ± 1.19	-0.47 ± 1.68	<0.01/<0.01	0.04
**FGM (mm) of sites for 16S RNA sequencing**	2.54 ± 0.52	0.47 ± 1.60	0.00 ± 1.96	<0.01/<0.01	0.29
**Presence of BOP at sites for metabololipidomics**	1/13	14/15	14/15	<0.01/<0.01	1.00
**Presence of BOP at sites for qRT-PCR**	3/13	14/15	15/15	<0.01/<0.01	0.32
**Presence of BOP at sites for 16S RNA sequencing**	1/13	14/15	14/15	<0.01/<0.01	1.00

PD, probing depth; CAL, clinical attachment level; FGM, level of free gingival margin; negative value indicates gingival recession (gingival margin is below cementoenamel junction); BOP, bleeding on probing; NA, not available due to the subjects are the same in the P and A groups; p-values were calculated using Student’s t-test, paired t-test, Chi-squared test or McNemar test.

**Figure 1:**
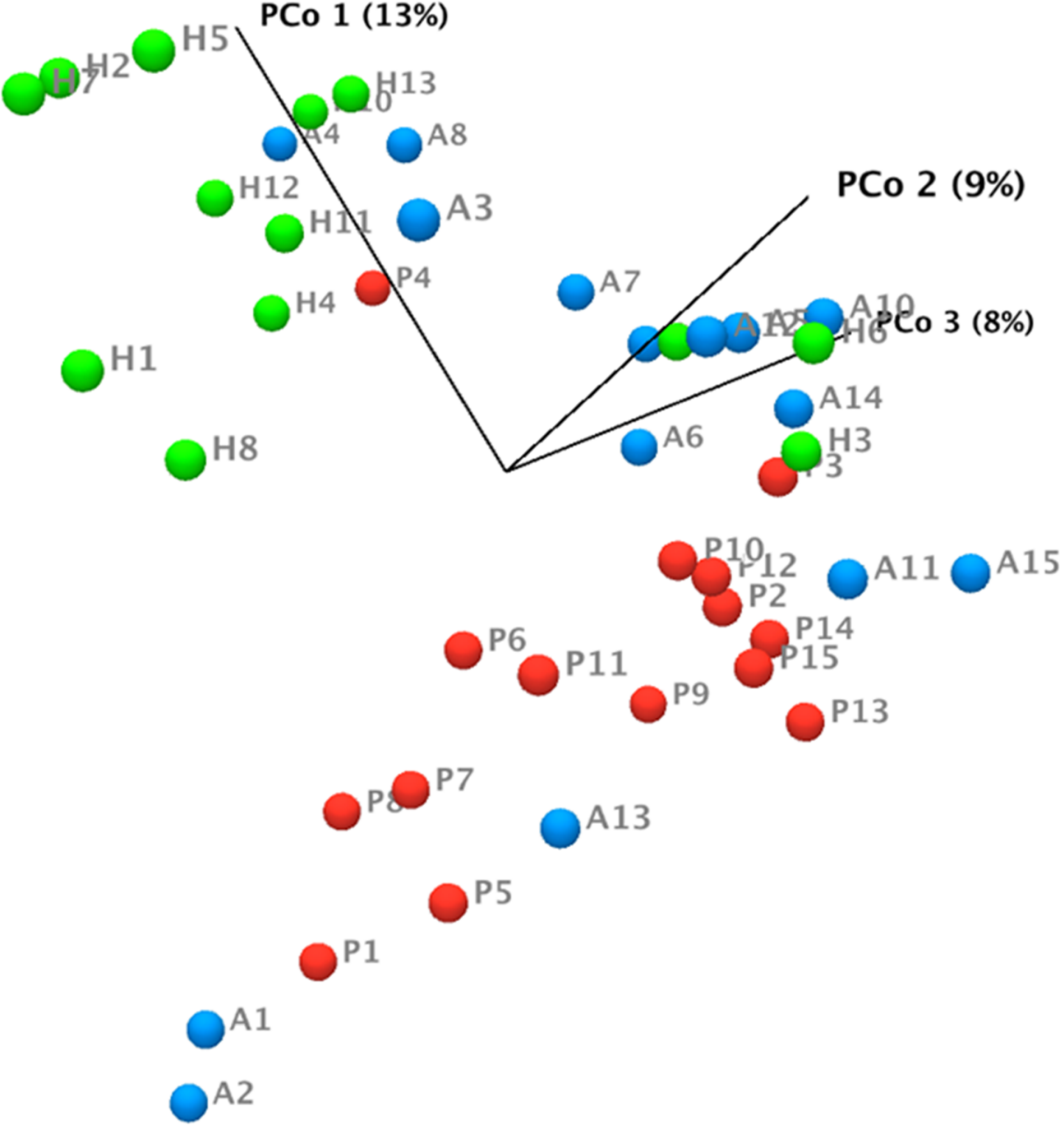
Bray-Curtis principal coordinate analysis (PCoA) plot for subgingival microbiome profiles. Axis one accounts for 13% of sample variance, while axis two and three account for 9% and 8% of variance, respectively. One dot represents one sample in each group. This plot demonstrates clusters of samples based on their similarity of microbial composition. These three groups display distinct microbial compositions. (H (green): healthy; P (red): periodontitis before non-surgical therapy; A (blue): periodontitis after non-surgical therapy).

**Figure 2:**
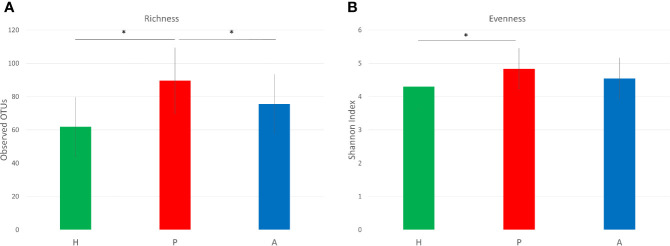
Alpha diversity of the subgingival microbiome. **(A)** Richness of the subgingival microbiome. Richness is represented by the number of operational taxonomic units (OTUs). **(B)** Evenness of the subgingival microbiome. Evenness is represented by Shannon index. (*p < 0.05; H (green): healthy; P (red): periodontitis before non-surgical therapy; A (blue): periodontitis after non-surgical therapy).

Three hundred and sixty one OTUs were identified across all samples. The beta-diversity, which represents the variation in microbial composition between groups, was significantly different between the healthy group and the periodontitis prior to SRP group as well as between the healthy group and the periodontitis after SRP group (p=<0.01,<0.01, respectively; **Figure 1**). Significance was not seen between the periodontitis prior to SRP group and the periodontitis after SRP group (p= 0.57), suggesting periodontal treatment did not entirely change the microbial composition to a healthy condition. The bacterial species richness (the number of OTUs) in the periodontitis prior to SRP group was significantly higher than in the healthy and periodontitis after SRP groups (p=<0.01, 0.03 respectively, **Figure 2A**). The mean Shannon index (evenness) was the highest in the periodontitis prior to SRP group and lowest in the healthy group. There was only a significant difference in bacterial species evenness between the periodontitis prior to SRP and healthy groups (p=0.04, **Figure 2B**). These results indicate that species were the most diverse in periodontitis before treatment.

Considering the OTU species assignments, *Corynebacterium matruchotii*, *Fusobacterium nucleatum*_subsp._*animalis*, *Fusobacterium nucleatum*_subsp._*polymorphum*, *Fusobacterium nucleatum*_subsp._*vincentii*, and *Veillonella dispar*, were seen in all three groups with varying abundance (**Table 2**). The top three species that had the highest relative abundance in the healthy group were *Neisseria oralis* (10%), *Corynebacterium matruchotii* (6%), and *Actinomyces* sp._oral_taxon_169 (5%). Periodontitis sites before and after therapy were dominated by *Fusobacterium nucleatum* subsp. *vincentii* at 8% and 6%, respectively. While considering species with relative abundance ≥1%, three putative red complex periodontal pathogens, *Porphyromonas gingivalis*, *Tannerella forsythia* and *Treponema denticola* were present in the periodontitis prior to SRP group and *Porphyromonas gingivalis* and *Treponema denticola* were present in the periodontitis after SRP group. These species had very low abundance and were infrequently present in the healthy group.

**Table 2:** Relative abundance of bacterial species.

H: Healthy		P: Periodontitis before non-surgical therapy		A: Periodontitis after non-surgical therapy
**Bacterial species**	**Relative abundance**	**Bacterial species**	**Relative abundance**	**Bacterial species**	**Relative abundance**
*Neisseria oralis*	0.10	*Fusobacterium nucleatum_*subsp._*vincentii*	0.08	*Fusobacterium nucleatum_*subsp._*vincentii*	0.06
*Corynebacterium matruchotii*	0.06	*Escherichia coli*	0.06	*Corynebacterium matruchotii*	0.04
*Actinomyces* sp._oral_taxon_169	0.05	*Porphyromonas gingivalis*	0.05	*Fusobacterium nucleatum_*subsp._*polymorphum*	0.04
*Fusobacterium nucleatum_*subsp._*animalis*	0.04	*Bifidobacterium longum*	0.05	*Prevotella nigrescens*	0.04
*Campylobacter gracilis*	0.04	*Treponema denticola*	0.03	*Rothia dentocariosa*	0.04
*Actinomyces naeslundii*	0.04	*Corynebacterium matruchotii*	0.02	*Fusobacterium nucleatum*_subsp._*animalis*	0.03
*Actinobaculum* sp._oral_taxon_183	0.04	*Fusobacterium nucleatum_*subsp._*animalis*	0.02	*Actinomyces* sp._oral_taxon_169	0.03
*Fusobacterium nucleatum_*subsp._*vincentii*	0.03	*Fusobacterium nucleatum_*subsp._*polymorphum*	0.02	*Neisseria pharyngis*	0.03
*Rothia dentocariosa*	0.03	*Prevotella nigrescens*	0.02	*Porphyromonas gingivalis*	0.02
*Veillonella dispar*	0.03	*Veillonella dispar*	0.02	*Treponema denticola*	0.02
*Lautropia mirabilis*	0.03	*Porphyromonas endodontalis*	0.02	*Veillonella dispar*	0.02
*Rothia aeria*	0.03	*Leptotrichia buccalis*	0.02	*Porphyromonas endodontalis*	0.02
*Fusobacterium nucleatum_*subsp._*polymorphum*	0.02	*Fusobacterium* sp._oral_taxon_203	0.02	*Campylobacter gracilis*	0.02
*Haemophilus parainfluenzae*	0.02	*Bacteroidales_[G-2]* sp._oral_taxon_274	0.02	*Selenomonas noxia*	0.02
*Actinomyces* sp._oral_taxon_171	0.02	*Tannerella forsythia*	0.02	*Actinomyces* sp._oral_taxon_170	0.02
*Actinomyces massiliensis*	0.02	*Veillonella parvula*	0.02	*Streptococcus gordonii*	0.02
*Fusobacterium naviforme*	0.02	*Bacteroidaceae_[G-1]* sp._oral_taxon_272	0.02	*Capnocytophaga leadbetteri*	0.02
*Corynebacterium durum*	0.02	*Campylobacter gracilis*	0.01	*Streptococcus* sp._oral_taxon_058	0.01
*Selenomonas noxia*	0.01	*Streptococcus* sp._oral_taxon_058	0.01	*Campylobacter rectus*	0.01
*Actinomyces* sp._oral_taxon_170	0.01	*Prevotella intermedia*	0.01	*Neisseria oralis*	0.01

These bacterial species are listed according to their mean relative abundance ranging from high to low in each group. Only twenty species with the highest relative abundance in each group are listed. The relative abundance ranges from 0.01 to 1 (1 to 100%).

**Figure 3:**
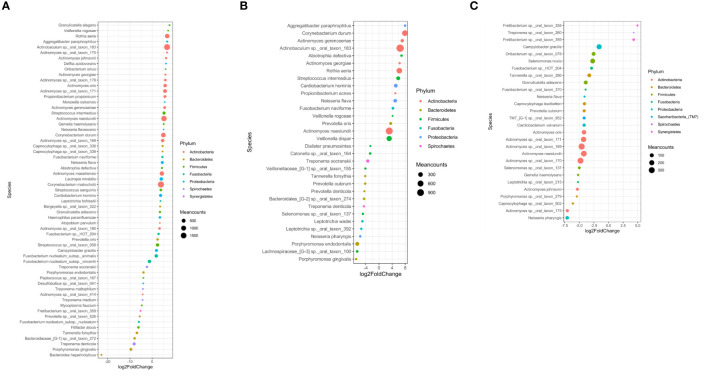
Differentially abundant operational taxonomic units (OTUs) in three comparisons. Each circle represents one OTU (bacterial species). The OTUs are color-coded based on the phylum to which they belong and plotted based on their log2 fold change in each comparison. The size of the circle is proportional to the mean count of each species. All differentially abundant OTUs in each comparison are listed. **(A)** Healthy (H) *vs*. Periodontitis before non-surgical therapy (P); **(B)** Healthy (H) *vs*. Periodontitis after non-surgical therapy (A); **(C)** Periodontitis before non-surgical therapy (P) *vs*. Periodontitis after non-surgical therapy (A). In these comparisons, the second group is the reference group. The fold change is calculated by dividing the bacterial abundance in the first group by the bacterial abundance in the second group (H/P, H/A, P/A respectively). These graphs demonstrate different microbial compositions between the three groups.

Among two-group comparisons, there were 61 bacterial species with significant differences in relative fold change between the healthy and periodontitis prior to SRP groups, 32 bacterial species with significant differences in relative fold change between the healthy and periodontitis after SRP groups and 28 bacterial species with significant differences in relative fold change between the periodontitis prior to SRP and after SRP groups (**Figures 3A–C** and **Supplementary Table 2**). The relative amounts of *P. gingivalis*, *T. denticola*, and *T. forsythia* in both the periodontitis prior to SRP and after SRP groups were significantly higher than in the healthy group (**Figures 3A, B**), but there was no significant difference between the two periodontitis groups. Periodontal health related species, *Rothia aeria* and *Corynebacterium durum*, in the healthy group had significantly higher relative fold values than the two periodontitis groups.

### Correlations Between Lipid Mediator, SPM Receptor Gene and Subgingival Microbiome

**Figure 4:**
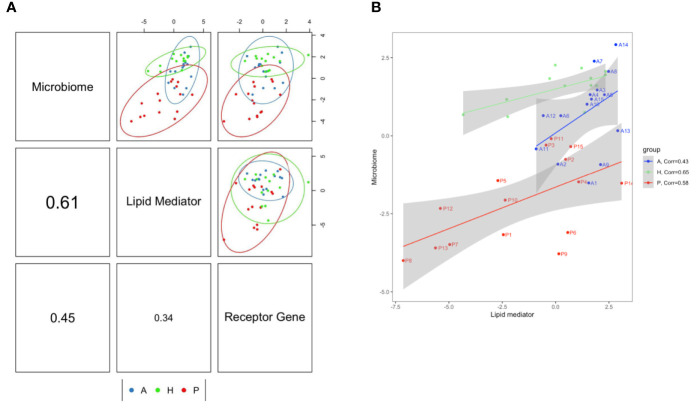
Correlations between lipid mediator profile, specialized pro-resolving lipid mediator (SPM) receptor gene profile and subgingival microbiome profile. **(A)** The component correlation plots represent subgingival microbiome-lipid mediator profile (upper-middle box), subgingival microbiome-receptor gene profile (upper-right box), and lipid mediator-receptor gene profile (middle-right box). The correlation coefficient of these profiles: subgingival microbiome-lipid mediator (middle-left box)= 0.61; subgingival microbiome-receptor gene (lower-left box)= 0.45; lipid mediator-receptor gene (lower-middle box)= 0.34. **(B)** The correlation plot demonstrates the subgingival microbiome-lipid mediator correlation patterns in each subject group. The correlation coefficients for the H, P and A groups are 0.65, 0.58 and 0.43, respectively. Most of the subject’s subgingival microbiome-lipid mediator profiles (subjects are labeled in the plot) in periodontitis before treatment move toward the healthy patterns after treatment. (H (green): healthy; P (red): periodontitis before non-surgical therapy; A (blue): periodontitis after non-surgical therapy; one dot represents one subject in each group; numbers on axes represent relative levels of bacterial species, lipid mediators or receptor gene expression; the analyses are performed using the DIABLO method.)

Previously, we demonstrated that the level of six lipid mediators (5-HETE, 15-HETE, 15(S)-HEPE, 4-HDHA, 7-HDHA, 17-HDHA) and expression of D-series resolvins corresponding receptor genes (*GPR18*, *GPR32*) were significantly altered by periodontitis treatment (21). In the present study, data of lipid mediator levels, receptor gene expression and bacterial species abundance were integrated and analyzed by the DIABLO method to demonstrate profile correlations. When all samples from the three subject groups are included, the correlation coefficient between subgingival microbiome and lipid mediator profiles was 0.61, the correlation coefficient between subgingival microbiome and receptor gene profiles was 0.45 and the correlation coefficient between lipid mediator and receptor gene profiles was 0.34. (**Figure 4A**). As the results at the species level, the correlation between subgingival microbiome and lipid mediator profiles was also the highest at the genus level. (correlation coefficient= 0.5) (**Supplementary Figure 1A**). Since the subgingival microbiome and lipid mediator profiles had the highest correlation, the subgingival microbiome-lipid mediator profile correlation was further assessed in each group and the correlation coefficients were 0.65, 0.58 and 0.43 in the healthy, periodontitis prior to SRP and periodontitis after SRP groups respectively (**Figure 4B**). The correlation patterns at the genus level in each group were also similar to those at the species level (**Supplementary Figure 1B**).

**Figure 5:**
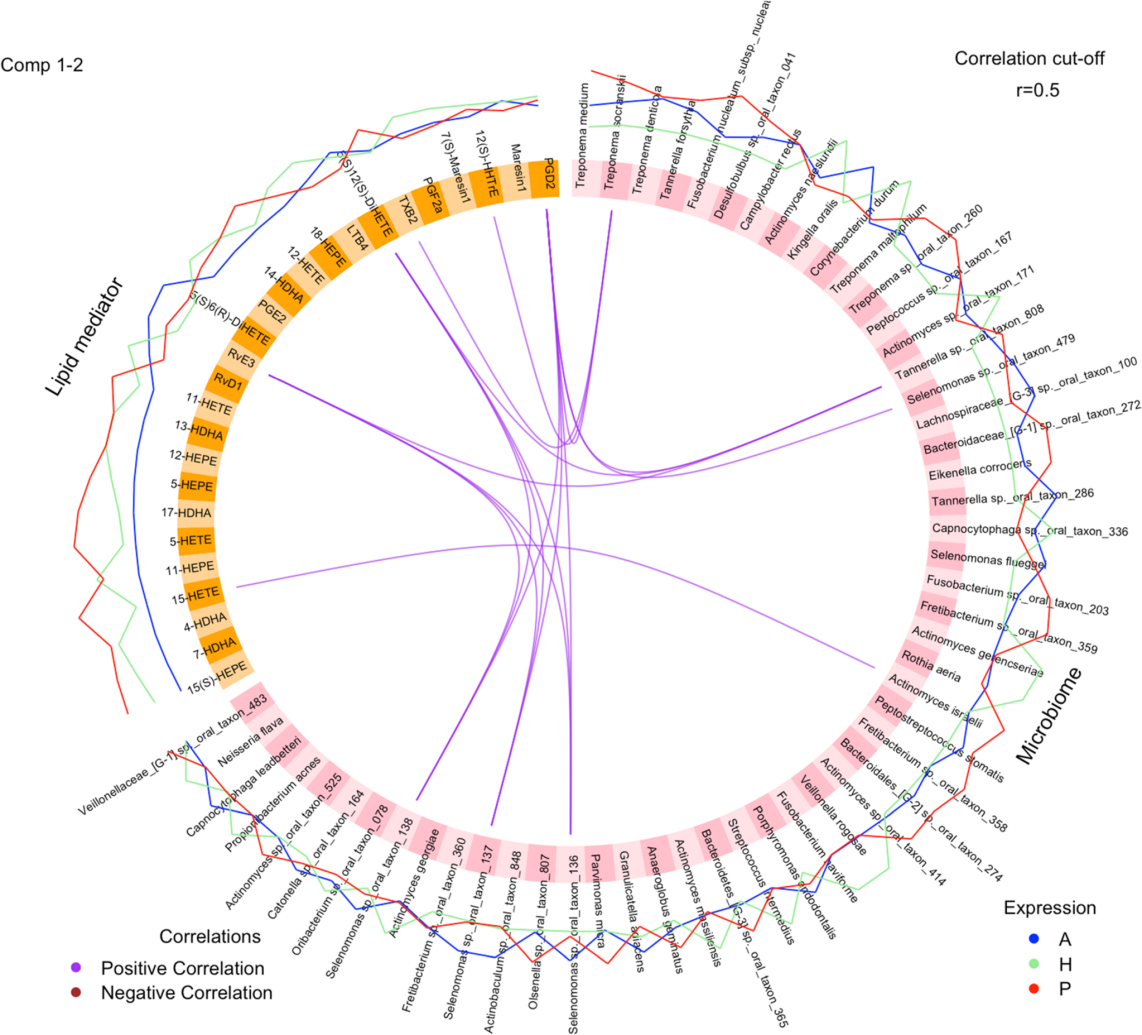
Correlations between individual lipid mediators and bacterial species. The circos plot shows correlations between lipid mediators (yellow) and bacterial species (pink). Three lines outside the circle represent the relative levels of lipid mediators or bacterial species (H (green): healthy; P (red): periodontitis before non-surgical therapy; A (blue): periodontitis after non-surgical therapy). Purple lines inside the circle represent positive correlations between lipid mediators and bacterial species with correlation coefficient ≥0.5. None of the bacterial species and lipid mediators has a significant negative correlation coefficient ≤-0.5. The analysis is performed using the DIABLO method.

**Figure 6:**
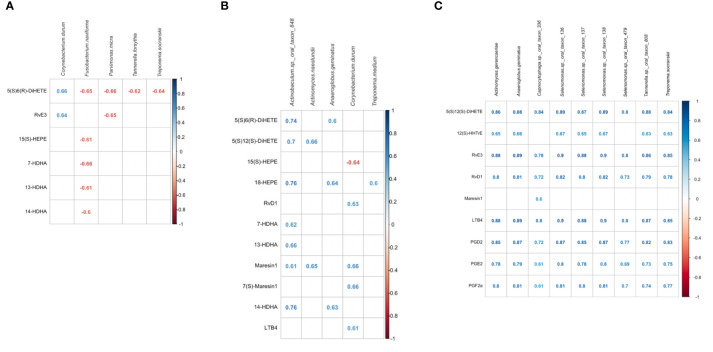
Correlations between bacterial species and lipid mediators in the three groups. **(A)** correlated lipid mediators and bacterial species in the healthy group. **(B)** correlated lipid mediators and bacterial species in the periodontitis before non-surgical therapy group. **(C)** correlated lipid mediators and bacterial species in the periodontitis after non-surgical therapy group. The analysis is performed using the Sparse Partial Least Squares (SPLS) method. Bacterial species are listed in alphabetical order. Lipid mediators are grouped by biosynthetic pathways; leukotriene pathway marker: 5(S),6(R)-DiHETE; lipoxygenase pathway marker: 5(S),12(S)-DiHETE; prostaglandin pathway marker: 12(S)-HHTrE; E-series resolvins and pathway markers (derived from omega-3 EPA): 15(S)-HEPE, 18-HEPE, RvE3; D-series resolvins and pathway markers (derived from omega-3 DHA or DPA): RvD1, 7-HDHA, 13-HDHA; Maresins and pathway markers (derived from omega-3 DHA or DPA): 14-HDHA, MaR1, 7(S)-MaR1; pro-inflammatory lipid mediators: LTB4, PGD2, PGE2, PGF2a. Some pathway markers are involved in multiple pathways but only one major pathway is listed. All correlated lipid mediators-bacterial species have absolute correlation coefficients ≥0.60.

Specific bacterial species and lipid mediators were correlated (correlation coefficient ≥0.5) while including all samples of the three groups (**Figure 5**). Then, SPLS analysis was conducted to identify individual bacterial species and lipid mediators contributing to the subgingival microbiome-lipid mediator profile correlation in each group. Using a correlation coefficient cut off of ≥0.6, five bacterial species and six lipid mediators were correlated in the healthy group, five bacterial species and 11 lipid mediators were correlated in the periodontitis prior to SRP group and nine bacterial species and nine lipid mediators were correlated in the periodontitis after SRP group (**Supplementary Table 3** and **Figure 6**). These correlations indicate that the relative abundance of these bacteria species and the level of these lipid mediators are highly associated.

When comparing these correlated species in periodontitis before treatment to healthy controls, only *Corynebacterium durum*, a periodontal health related species, was identified in both conditions, but correlated with different lipids. In health, *Corynebacterium durum* was positively correlated with the leukotriene pathway marker, 5(S)6(R)-DiHETE, and one of the E-series resolvins, RvE3. In periodontitis before treatment, *Corynebacterium durum* was negatively correlated with the resolvin pathway marker, 15(S)-HEPE, and positively correlated with the proinflammatory mediator, LTB4, and SPMs, including 7(S)-Maresin1, Maresin1 and RvD1. Both conditions had five lipid mediators, 5(S)6(R)-DiHETE, 15(S)-HEPE, 7-HDHA, 13-HDHA and 14-HDHA, correlated with different bacterial species.

When comparing these correlated species in periodontitis after treatment to healthy controls, *Treponema socranskii*, a periodontal pathogen, was identified in both conditions. This bacterial species was negatively correlated to 5(S)6(R)-DiHETE in the healthy group and positively correlated with different lipids in the periodontitis after SRP group. The E-series resolvin, RvE3, was the only lipid mediator identified in both conditions.

When comparing these correlated species in periodontitis before treatment to after treatment, a bacterial species, *Anaeroglobus geminatus*, was identified in both conditions that positively correlated with different lipid mediators. Both conditions had four lipid mediators, 5(S)12(S)-DiHETE, RvD1, Maresin 1 and LTB4, correlated with different bacteria species. Among the nine bacterial species identified in the periodontitis after SRP group, four *Selenomonas* species (*Selenomonas* sp._oral_taxon_136, *Selenomonas* sp._oral_taxon_137, *Selenomonas* sp._oral_taxon_138, *Selenomonas* sp._oral_taxon_479) are highly correlated with multiple lipid mediators.

## Discussion

Many studies demonstrate that shifts in the bacterial load and bacterial composition within the subgingival microbiota are correlated with periodontal conditions (30, 31). Associations between periodontal inflammation and molecular-level host responses (32, 33) or metabolite profiles (34, 35) have also been investigated. However, there is a remarkable lack of studies assessing clinical host-microbiota relationship in periodontitis. The current study showed different correlations between levels of lipid mediators, expression of SPM receptor genes and abundance of subgingival bacterial species in health vs. disease. To the best of our knowledge, this is the first study demonstrating that the molecular profiles for lipid-mediated resolution of inflammation activity are associated with microbial composition shifts in human periodontitis. The correlations between specific lipid mediators and bacterial species indicate their potential interactions, which are important for deciphering the mechanisms of resolution of inflammation in the pathogenesis of periodontitis.

In this study, most of the periodontitis subjects have severe periodontitis (Stage III, Grade C). In addition to the lack of maintenance or poor self-care, these patients may have abnormal host responses causing severe, rapid periodontal destruction and dysbiotic subgingival microbiota. Current results indicate that failure of resolution associated with the imbalanced lipid mediator profiles could be the potential mechanisms (21). This study compared samples from the diseases sites of the periodontitis subjects to the healthy sites of the subjects without periodontitis because lipid mediator levels and profiles are distinct at the subject level (16, 17). However, it would be interesting to analyze samples from both diseases sites and healthy sites of the same periodontitis subjects to evaluate the subgingival microbiome-lipid mediator correlations at different local environments.

According to the profile correlation analysis, the correlation between lipid mediator profile and receptor gene profile was lower than both the subgingival microbiome-lipid mediator profile correlation and subgingival microbiome-receptor gene profile correlation. The low lipid mediator-receptor gene profile correlation supports the hypothesis that an imbalance between lipid mediator levels and receptor expression results in failure of resolution of inflammation in periodontitis (21). It is known that expression of SPM corresponding receptors is important for resolution of inflammation. Deficient expression of these receptors has been associated with increased inflammation in peritonitis (36, 37), paw edema (38), and microbial sepsis (39) in pre-clinical models. Overexpression of SPM receptors, such as ChemR23 (ERV1), was associated with immune responses favorable for inflammation resolution in a dorsal air pouch model (40).

As with the clustering in both lipid mediators and SPM receptor gene expression (21), the clusters of subgingival microbiome were associated with inflammatory conditions in gingiva (**Figure 1**). Current results of 16S rRNA sequencing show that periodontitis subjects had a higher abundance of periodontal pathogens and microbial diversity than healthy subjects. Healthy subjects also have a higher abundance of periodontal health related bacterial species. These findings were similar to published results in the literature (41–45).

The highest subgingival microbiome-lipid mediator profile correlation (**Figure 4A**) suggests that the composition of the subgingival microbiota could be significantly affected by periodontal inflammation mediated by SPMs and other relevant lipid mediators (13, 14). The correlation pattern in the periodontitis after SRP group was closer to the healthy group than the periodontitis prior to SRP group suggesting periodontal treatment moves profiles of lipid mediators and subgingival microbiota toward non-diseased profiles (**Figure 4B**). Non-surgical periodontal therapy not only improves clinical parameters but also changes molecular profiles closer to the homeostatic condition. It is possible that local debridement stimulates SPM-mediated resolution of inflammation influencing microbiota shifts in the re-establishment of the subgingival biofilm. In the correlation analysis between individual lipid mediators and bacterial species, most of the correlated lipid and bacterial species are specific for the subject group and not shared by other groups. These results also support that the subgingival microbiome and lipid mediator profiles are specifically associated with periodontal inflammatory conditions.

The correlation analysis identified some bacterial species infrequently discussed in the literature and several lipid mediators potentially important for resolution of periodontal inflammation (**Figure 6**). A periodontal health related bacterial species, *Corynebacterium durum*, had relatively low abundance in periodontitis, but its positive correlation with several SPMs indicate that gingival tissues produce SPMs to regulate periodontal inflammation resulting in the presence of this periodontal health related bacterial species. In periodontitis before treatment, one bacterial species, *Actinobaculum* sp._oral_taxon_848 (46), had the greatest number of correlated resolution related lipid mediators, 5(S)12(S)-DiHETE, 5(S)6(R)-DiHETE, 18-HEPE, 7-HDHA, 13-HDHA, 14-HDHA, Maresin1, and higher correlations than other species. A recently identified bacterial species, *Anaeroglobus geminatus* (47), related to periodontitis and rheumatoid arthritis, was identified in periodontitis before and after treatment, but was highly correlated with different lipid mediators in the two inflammatory conditions. These bacterial species could be important for periodontal inflammation and deserve further investigation.

*Selenomonas* species were dominant in the identified bacterial species correlated with multiple lipid mediators in the periodontitis after SRP group. Although *Selenomonas* species are not frequently discussed in the literature, some studies show associations between the presence, as well as abundance, of *Selenomonas* species and periodontitis (48–50), and the fundamental role of *Selenomonas* species in the structure of subgingival biofilm (51). Tanner and coworkers found that *Selenomonas noxia* was associated with the progression of periodontitis (52). In the current study, most of the identified *Selenomonas* species had the highest relative abundance in the periodontitis after SRP group although the relative abundance was generally low (<1%, **Supplementary Table 4**). The abundance of *Selenomonas* species was highly and positively associated with levels of two SPMs, RvE3 and RvD1 in periodontitis after treatment. An *in vitro* study investigating the impact of dietary lipid supplements on the microbiota in the rumen ecosystem showed that fish oil in the diet is associated with an increased amount of *Selenomonas* species in fermenters (53) suggesting omega-3 fatty acids influence the composition of *Selenomonas* species and other bacterial species. The current findings support the association between the abundance of *Selenomonas* species and levels of lipid mediators in resolution of periodontal inflammation following treatment. Although the exact role of *Selenomonas* species in the resolution of periodontal inflammation has to be further studied, *Selenomonas* species appear to be sensitive to the resolution of inflammation after treatment and the changes of their abundance could be critically associated with the changes of periodontal inflammatory conditions.

As with *Selenomonas* species, two periodontitis pathogens, *Treponema socranskii* (50, 54, 55) and *Tannerella* sp._oral_taxon_808 (56), and an oral biofilm early colonizer species, *Actinomyces gerencseriae*, were also correlated with RvE3, RvD1 and other lipid mediators, including proinflammatory mediators, LTB4, PGD2, PGE2 and PGF2a, in periodontitis after treatment. These results indicate that the resolution phase of periodontal inflammation induced by periodontal treatment includes a combination of proresolving and proinflammatory mediators highly correlated with non-periodontitis or periodontitis associated bacterial species, explaining the specific phase of inflammation resolution between untreated periodontitis and healthy phases.

We previously reported that levels of six lipid mediators in periodontitis before treatment were significantly higher than in periodontitis after treatment (21). In this study, among the six lipid mediators, only 15(S)-HEPE, a pathway marker for E-series resolvins, was negatively correlated with *Corynebacterium durum*, and 7-HDHA, a pathway marker for D-series resolvins, was positively correlated with *Actinobaculum* sp._oral_taxon_848 in periodontitis before treatment. These two SPM pathway markers appear to be more associated with the subgingival microbiota in periodontitis than the others. Two lipids derived from arachidonic acid, 5(S),12(S)-DiHETE and 5(S)6(R)-DiHETE, were correlated with multiple bacterial species in the three subject groups. 5(S),12(S)-DiHETE is an epimer of leukotriene B_4_ weakly chemotactic for neutrophils (57). 5(S)6(R)-DiHETE is a hydrolysis product of leukotriene A_4_ and can bind to the leukotriene receptor (58). The identification of these two lipids in the correlation analysis suggests their relevant synthetic pathways for leukotrienes could be actively associated with changes in the subgingival microbial composition.

Resolution of inflammation induced by SPMs is critical for periodontal regeneration and healing given chronic and excessive inflammation is detrimental to homeostasis (9). Several SPMs, including RvD1, RvE3, Maresin1 and 7(S)-Maresin 1, were identified in the current correlation analysis. Resolvins are known to resolve inflammation, stimulate periodontal tissue healing and regenerate lost alveolar bone. RvE1 induces periodontal regeneration in pre-clinical periodontitis models (12–14, 59, 60), promotes regenerative properties of periodontal ligament (PDL) stem cells (61), and regulates osteoclasts as well as osteoblasts favorably for bone preservation (62–64). RvE3 demonstrates potent resolution properties by limiting neutrophil infiltration in the peritonitis model and inhibiting neutrophil chemotaxis (65–67). RvD1 inhibits osteoclast differentiation and reduces bone destruction in an arthritis preclinical model (68), inhibits production of pro-inflammatory cytokines from gingival fibroblasts (69) and enhances PDL fibroblast proliferation as well as wound closure (70). Maresins can promote periodontal tissue healing and regulate periodontal inflammation. MaR1 promotes survival and inhibits apoptosis in PDL cells (71), induces regenerative properties of PDL stem cells (61), and restores phagocytic capacity of macrophages from periodontitis patients (72). Both RvD1 and RvE3 were highly correlated with multiple bacterial species in periodontitis after treatment suggesting their important role in resolution of periodontal inflammation associated with subgingival microbiota shift. Their mean levels also increased in periodontitis after treatment as compared to periodontitis before treatment (21). According to the current findings and molecular actions described in the literature, RvD1 and RvE3 potentially could be utilized to treat periodontitis.

In general, results of the correlation analyses indicate significant associations between lipid mediator levels and subgingival bacterial abundance in periodontal inflammation. It is possible that immune responses mediated by SPMs and other lipid mediators drive the shifts of the subgingival microbiota, which can be supported by preclinical models showing oral microbiota shifts following the local administration of SPMs (13, 14). According to *in vitro* experiments, SPMs do not have direct anti-bacterial properties (73). However, the other direct effects of these lipid mediators on bacterial growth or other activities could not be excluded (74). On the other hand, oral bacteria might affect lipid synthesis and metabolism. It is known that the metabolites from gut microbiota affects host lipid metabolism and lipid composition (75). Oral microbiota could do the same. Additionally, periodontal tissues could produce more lipid mediators to regulate excessive inflammation induced by the increased amount of bacteria in periodontitis (73). These potential mechanisms for subgingival microbiome-lipid mediator correlations have to be further studied. All of these possibilities indicate the important role of lipid mediator profiles in dysbiosis of the subgingival microbiota in periodontitis.

With some limitations, current results should be carefully interpreted. Profiles of lipid mediators and receptor genes were derived from gingival tissues. Since lipid mediators can be produced by and receptor genes can be expressed on a variety of cells, including neutrophils, macrophages, fibroblasts, osteoblasts and osteoclasts (14, 62–64, 69, 70, 76), these molecular profiles should be interpreted as a snapshot of inflammatory reactions in gingiva, but cannot fully explain cellular activities. Additionally, results of the correlation analysis could be biased by the limited number of subjects and subject variability. It is necessary to conduct *in vitro* or *in vivo* experiments to further investigate these correlations and potential interactions between bacterial species and lipid mediators.

In conclusion, profiles of lipid mediators, receptor genes and the subgingival microbiome were distinct in different periodontal inflammatory conditions. The highly correlated lipid mediator and subgingival microbiome profiles and specific correlations between individual lipid mediators and bacterial species indicate that periodontal inflammation regulated by lipid mediators drives the shifts of the subgingival microbiota. Elucidation of these correlations facilitates the understanding of resolution of inflammation in periodontitis and identification of potential biological targets for the development of novel therapies.

## Data Availability Statement

The datasets presented in this study can be found in online repositories. The names of the repository/repositories and accession number(s) can be found below: NCBI BioProject ID: PRJNA726254.

## Ethics Statement

The studies involving human participants were reviewed and approved by UTHealth Committee for the Protection of Human Subjects. The patients/participants provided their written informed consent to participate in this study.

## Author Contributions

C-TL and TD contributed conception and design of the study. C-TL and BF collected clinical samples. C-TL, GT and BF processed clinical samples and performed experiments. C-TL, RL, LZ, GT, WZ and KM performed the data analysis. C-TL, RL, LZ, GT, WZ, KM, NA, and TD contributed to data interpretation. C-TL wrote the first draft of the manuscript. All authors wrote sections of the manuscript. All authors contributed to the article and approved the submitted version.

## Funding

This work was supported in part by UTHealth School of Dentistry Grants Program in Dental Research (C-TL), UTHealth the Center for Clinical and Translational Sciences (CCTS) Translational Technologies Core Laboratories Awards (C-TL), USPHS grant DE025020 from the National Institute of Dental and Craniofacial Research (TD), CPRIT RP170668 (WZ) and NIH 1 UL1 TR003167 01 (WZ).

## Conflict of Interest

The authors declare that the research was conducted in the absence of any commercial or financial relationships that could be construed as a potential conflict of interest.

## Abbreviations

5(S),6(R)-DiHETE, 5(S),6(R)-dihydroxyeicosatetraenoic acid; 5(S),12(S)-DiHETE, 5(S),12(S)-dihydroxy-*6E,8Z,11E,14Z*-eicosatetraenoic acid; 7-HDHA, 7-hydroxydocosahexaenoic acid; 12(S)-HHTrE, 12(S)-hydroxyheptadecatrienoic acid; 13-HDHA, 13-hydroxydocosahexaenoic acid; 14-HDHA, 14-hydroxydocosahexaenoic acid; 15(S)-HEPE, 15(S)-hydroxyeicosapentaenoic acid; 18-HEPE, 18-hydroxyeicosapentaenoic acid; DHA, docosahexaenoic acid; DPA, docosapentaenoic acid; EDTA, ethylenediaminetetraacetic acid; EPA, eicosapentaenoic acid; LTB4, leukotriene B4; MaR1, maresin 1, 7(S)-MaR1, 7-epi maresin 1; PGD2, prostaglandin D2; PGE2, prostaglandin E2; PGF2a, prostaglandin F2a; RvD1, resolvin D1; RvE3, resolvin E3.
