# Supplementary material for: Subgingival Microbiome and Specialized Pro-Resolving Lipid Mediator Pathway Profiles Are Correlated in Periodontal Inflammation
Source: Front Immunol. 2021 Jun 10;12:691216. doi: 10.3389/fimmu.2021.691216 (PMC8222734; doi:10.3389/fimmu.2021.691216)
Supplement: Supplementary file 1 [file Image_1.pdf]

Supplementary Figure 1. Correlations between lipid mediator profile, specialized pro-resolving lipid mediator (SPM) receptor gene profile and subgingival microbiome profile at the genus level. (A) The component correlation plots represent subgingival microbiome-lipid mediator profile (upper-middle box), subgingival microbiome-receptor gene profile (upper-right box), and lipid mediator-receptor gene profile (middle-right box). The correlation coefficient of these profiles: subgingival microbiome-lipid mediator (middle-left box)= 0.50; subgingival microbiome-receptor gene (lower-left box)= 0.41; lipid mediator-receptor gene (lower-middle box)= 0.36. **(B)** The correlation plot demonstrates the subgingival microbiome-lipid mediator correlation patterns in each subject group. The correlation coefficients for the H, P and A groups are 0.75, 0.43 and 0.42, respectively. Many of the subject's subgingival microbiome-lipid mediator profiles (subjects are labeled in the plot) in periodontitis before treatment move toward the healthy patterns after treatment. (H (green): healthy; P (red): periodontitis before non-surgical therapy; A (blue): periodontitis after non-surgical therapy; one dot represents one subject's profile in each group; numbers on axes represent relative levels of bacterial species, lipid mediators or receptor gene expression; the analyses are performed using the DIABLO method.)

(A)

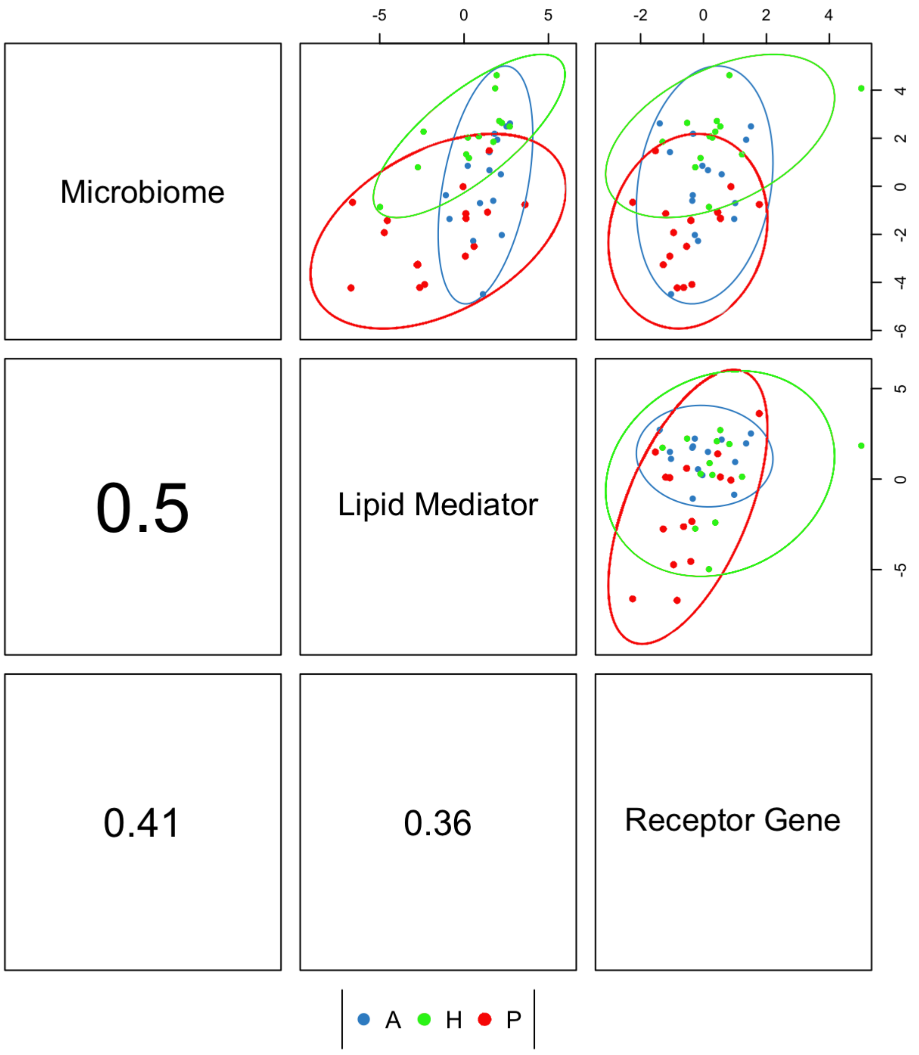

(B)

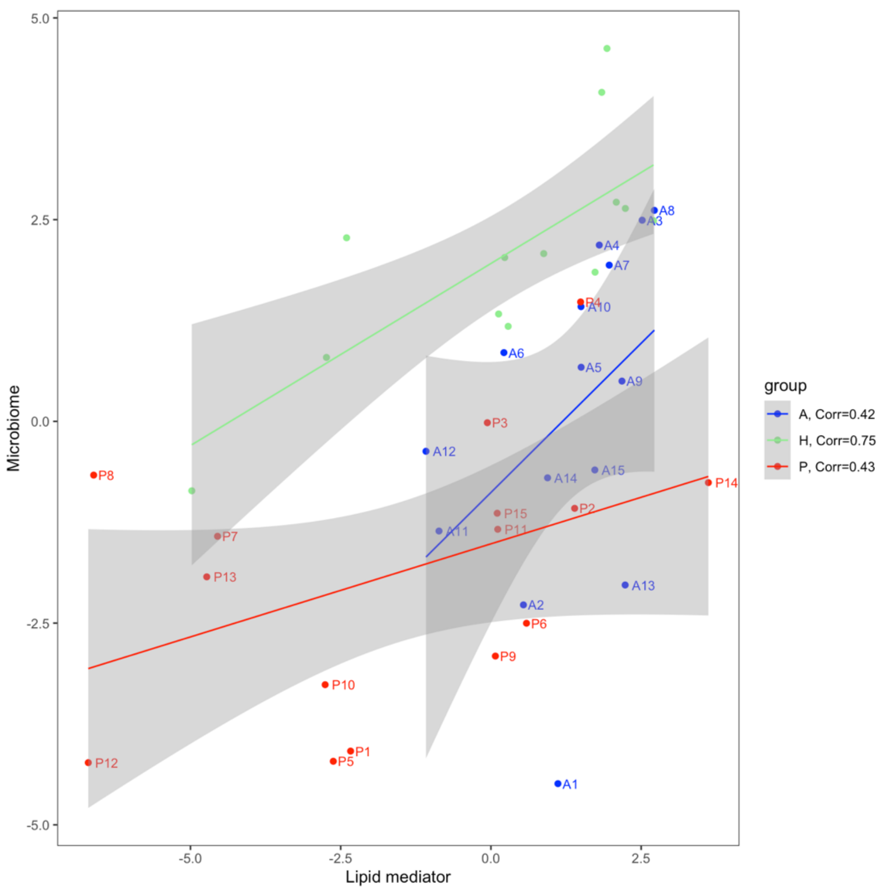

5.0
2.5
0.0
-2.5
-5.0
Microbiome
-5.0
-2.5
0.0
2.5
Lipid mediator
A8
A3
A4
A7
P4
A10
A6
A5
A9
P3
A12
A14
A15
P8
P14
P2
P15
P11
A11
P7
P13
A13
A2
P6
P9
P10
P1
P5
P12
A1
group
A, Corr=0.42
H, Corr=0.75
P, Corr=0.43
