# Supplementary material for: Subgingival Microbiome and Specialized Pro-Resolving Lipid Mediator Pathway Profiles Are Correlated in Periodontal Inflammation
Source: Front Immunol. 2021 Jun 10;12:691216. doi: 10.3389/fimmu.2021.691216 (PMC8222734; doi:10.3389/fimmu.2021.691216)
Supplement: Supplementary file 2 [file Table_1.docx]

**Supplementary Table 1. Diagnosis of the periodontitis subjects included in the study.**

| Extent | Severity | Grade | Case number |
| --- | --- | --- | --- |
| Generalized | Stage II | B | 1 |
| Localized | Stage III | B | 1 |
| Localized | Stage III | C | 1 |
| Generalized | Stage III | C | 12 |
