# Supplementary material for: Subgingival Microbiome and Specialized Pro-Resolving Lipid Mediator Pathway Profiles Are Correlated in Periodontal Inflammation
Source: Front Immunol. 2021 Jun 10;12:691216. doi: 10.3389/fimmu.2021.691216 (PMC8222734; doi:10.3389/fimmu.2021.691216)
Supplement: Supplementary file 3 [file Table_2.docx]

**Supplementary Table 2. Relative abundance difference of bacterial species between the three groups.**  These bacterial species have significantly different relative abundance between the groups (p-value<0.05; the p-values are adjusted by Benjamini–Hochberg procedure.) In these two-group comparisons, the second group is the reference group. The mean relative fold change is calculated by dividing the bacterial abundance in the first group by the bacterial abundance in the second group. These bacterial species are listed by the order of relative fold change (from the highest to the lowest). Only the bacterial species with the 10 highest or 10 lowest mean relative fold change are listed. In the comparison between the P and A groups, bacterial species with the 3 highest and 17 lowest mean relative fold change are listed because only three species have positive relative fold change. (H: Healthy; P: Periodontitis before non-surgical therapy; A: Periodontitis after non-surgical therapy).

| **H vs. P** | | | **H vs. A** | | | **P vs. A** | | |
| --- | --- | --- | --- | --- | --- | --- | --- | --- |
| **Bacterial Species** | **Mean fold change (log2)** | **Adjusted p-value** | **Bacterial Species** | **Mean fold change (log2)** | **Adjusted p-value** | **Bacterial Species** | **Mean fold change (log2)** | **Adjusted p-value** |
| *Granulicatella, elegans* | 7.77 | 0.0152 | *Aggregatibacter paraphrophilus* | 7.94 | 0.00930 | *Fretibacterium* sp._oral_taxon_358 | 4.95 | 0.0358 |
| *Veillonella rogosae* | 7.17 | 1.68E-04 | *Corynebacterium durum* | 7.89 | 2.29E-04 | *Treponema* sp._oral_taxon_260 | 4.31 | 0.0260 |
| *Rothia aeria* | 6.75 | 3.31E-05 | *Actinomyces gerencseriae* | 7.09 | 1.37E-05 | *Fretibacterium* sp._oral_taxon_359 | 4.30 | 0.0285 |
| *Aggregatibacter paraphrophilus* | 6.72 | 0.00755 | *Abiotrophia defectiva* | 6.87 | 0.00149 | *Capnocytophaga leadbetteri* | -4.11 | 0.00317 |
| *Actinobaculum* sp._oral_taxon_183 | 6.69 | 2.79E-05 | *Actinobaculum,* sp._oral_taxon_183 | 6.44 | 4.83E-04 | *Prevotella oulorum* | -4.16 | 0.0285 |
| *Actinomyces* sp._oral_taxon_175 | 6.66 | 5.38E-07 | *Actinomyces georgiae* | 6.28 | 0.00149 | *TM7_[G-1]* sp._oral_taxon_952 | -4.17 | 0.0352 |
| *Actinomyces johnsonii* | 6.24 | 8.28E-08 | *Rothia aeria* | 6.20 | 2.57E-05 | *Cardiobacterium* *valvarum* | -4.17 | 0.0316 |
| *Delftia acidovorans* | 6.20 | 0.00174 | *Streptococcus intermedius* | 5.85 | 2.57E-05 | *Actinomyces oris* | -4.19 | 9.54E-04 |
| *Oribacterium sinus* | 6.06 | 0.0462 | *Cardiobacterium hominis* | 5.12 | 0.00149 | *Actinomyces naeslundii* | -4.21 | 2.55E-04 |
| *Actinomyces georgiae* | 5.96 | 1.61E-05 | *Propionibacterium acnes* | 4.98 | 0.0232 | *Actinomyces* sp._oral_taxon_171 | -4.26 | 0.00317 |
| *Mycoplasma faucium* | -4.67 | 0.0189 | *Prevotella denticola* | -4.43 | 0.0364 | *Actinomyces* sp._oral_taxon_169 | -4.59 | 1.38E-04 |
| *Fretibacterium* sp._oral_taxon_359 | -5.21 | 0.0454 | *Bacteroidales_[G-2]* sp._oral_taxon_274 | -4.52 | 0.0128 | *Actinomyces* sp._oral_taxon_170 | -4.79 | 9.54E-04 |
| *Prevotella* sp._oral_taxon_526 | -5.59 | 0.0124 | *Treponema denticola* | -4.59 | 0.00400 | *Selenomonas* sp._oral_taxon_137 | -4.99 | 0.00379 |
| *Fusobacterium nucleatum_*subsp._nucleatum | -5.96 | 3.89E-05 | *Selenomonas* sp._oral_taxon_137 | -4.87 | 0.00749 | *Gemella haemolysans* | -5.20 | 0.0287 |
| *Filifactor alocis* | -6.17 | 6.19E-04 | *Leptotrichia wadei* | -5.03 | 0.0182 | *Leptotrichia* sp._oral_taxon_215 | -5.22 | 0.00379 |
| *Tannerella forsythia* | -6.86 | 2.46E-08 | *Leptotrichia* sp._oral_taxon_392 | -5.36 | 0.0217 | *Actinomyces johnsonii* | -5.32 | 1.10E-04 |
| *Bacteroidaceae_[G-1]* sp._oral_taxon_272 | -7.88 | 8.84E-07 | *Neisseria pharyngis* | -5.68 | 0.0427 | *Porphyromonas* sp._oral_taxon_279 | -5.41 | 0.0128 |
| *Treponema, denticola* | -8.10 | 5.94E-11 | *Porphyromonas endodontalis* | -6.59 | 0.00149 | *Capnocytophaga* sp._oral_taxon_902 | -6.09 | 0.0103 |
| *Porphyromonas gingivalis* | -9.54 | 2.31E-05 | *Lachnospiraceae_[G-3]* sp._oral_taxon_100 | -6.62 | 0.00153 | *Actinomyces* sp._oral_taxon_175 | -7.04 | 1.38E-04 |
| *Bacteroides heparinolyticus* | -22.80 | 4.93E-13 | *Porphyromonas gingivalis* | -6.94 | 0.0164 | *Neisseria pharyngis* | -7.06 | 0.0189 |
