# Supplementary material for: Subgingival Microbiome and Specialized Pro-Resolving Lipid Mediator Pathway Profiles Are Correlated in Periodontal Inflammation
Source: Front Immunol. 2021 Jun 10;12:691216. doi: 10.3389/fimmu.2021.691216 (PMC8222734; doi:10.3389/fimmu.2021.691216)
Supplement: Supplementary file 4 [file Table_3.docx]

**Supplementary Table 3. Correlations between bacterial species and lipid mediators.
(A) Correlations between bacterial species and lipid mediators in the healthy group.** Bacterial species are listed in alphabetical order. Correlations were calculated using the Sparse Partial Least Squares (SPLS) method and listed from the most positive or the most negative. Only absolute correlation coefficients≥0.60 are listed.

| **Bacterial species** | **Lipid mediator** | **Synthetic pathway for the lipid mediator** | **Correlation coefficient** |
| --- | --- | --- | --- |
| *Corynebacterium durum* | 5(S)6(R)-DiHETE | Leukotrienes | 0.6559 |
| *Corynebacterium durum* | RvE3 | E-series resolvins | 0.6414 |
| *Fusobacterium naviforme* | 7-HDHA | D-series resolvins | -0.6626 |
| *Fusobacterium naviforme* | 5(S)6(R)-DiHETE | Leukotrienes | -0.6494 |
| *Fusobacterium naviforme* | 15(S)-HEPE | E-series resolvins | -0.6149 |
| *Fusobacterium naviforme* | 13-HDHA | D-series resolvins | -0.6144 |
| *Fusobacterium naviforme* | 14-HDHA | Maresins | -0.6024 |
| *Parvimonas micra* | 5(S)6(R)-DiHETE | Leukotrienes | -0.6625 |
| *Parvimonas micra* | RvE3 | E-series resolvins | -0.6464 |
| *Tannerella forsythia* | 5(S)6(R)-DiHETE | Leukotrienes | -0.6233 |
| *Treponema socranskii* | 5(S)6(R)-DiHETE | Leukotrienes | -0.6352 |

**(B) Correlations between bacterial species and lipid mediators in the periodontitis prior to scaling and root planing group.** Bacterial species are listed in alphabetical order. Correlations were calculated using the Sparse Partial Least Squares (SPLS) method and listed from the most positive or the most negative. Only absolute correlation coefficients≥0.60 are listed.

| **Bacterial species** | **Lipid mediator** | **Synthetic pathway for the lipid mediator** | **Correlation coefficient** |
| --- | --- | --- | --- |
| *Anaeroglobus geminatus* | 18-HEPE | E-series resolvins | 0.6354 |
| *Anaeroglobus geminatus* | 14-HDHA | Maresins | 0.6323 |
| *Anaeroglobus geminatus* | 5(S)6(R)-DiHETE | Leukotrienes | 0.6010 |
| *Actinomyces naeslundii* | 5(S)12(S)-DiHETE | Lipoxygenase pathway | 0.6584 |
| *Actinomyces naeslundii* | Maresin 1 | Maresins | 0.6520 |
| *Actinobaculum* sp._oral_taxon_848 | 18-HEPE | E-series resolvins | 0.7623 |
| *Actinobaculum* sp._oral_taxon_848 | 14-HDHA | Maresins | 0.7575 |
| *Actinobaculum* sp._oral_taxon_848 | 5(S)6(R)-DiHETE | Leukotrienes | 0.7357 |
| *Actinobaculum* sp._oral_taxon_848 | 5(S)12(S)-DiHETE | Lipoxygenase pathway | 0.7030 |
| *Actinobaculum* sp._oral_taxon_848 | 13-HDHA | D-series resolvins | 0.6564 |
| *Actinobaculum* sp._oral_taxon_848 | 7-HDHA | D-series resolvins | 0.6234 |
| *Actinobaculum* sp._oral_taxon_848 | Maresin 1 | Maresins | 0.6087 |
| *Corynebacterium durum* | 7(S)-Maresin 1 | Maresins | 0.6648 |
| *Corynebacterium durum* | Maresin 1 | Maresins | 0.6593 |
| *Corynebacterium durum* | RvD1 | D-series resolvins | 0.6268 |
| *Corynebacterium durum* | LTB4 | Leukotrienes | 0.6095 |
| *Corynebacterium durum* | 15(S)-HEPE | E-series resolvins | -0.6409 |
| *Treponema medium* | 18-HEPE | E-series resolvins | 0.6006 |

**(C) Correlations between bacterial species and lipid mediators in the periodontitis after scaling and root planing group.** Bacterial species are listed in alphabetical order. Correlations were calculated using the Sparse Partial Least Squares (SPLS) method and listed from the most positive or the most negative. Only absolute correlation coefficients≥0.60 are listed.

| **Bacterial species** | **Lipid mediator** | **Synthetic pathway for the lipid mediator** | **Correlation coefficient** |
| --- | --- | --- | --- |
| *Anaeroglobus geminatus* | RvE3 | E-series resolvins | 0.8936 |
| *Anaeroglobus geminatus* | LTB4 | Leukotrienes | 0.8918 |
| *Anaeroglobus geminatus* | 5(S)12(S)-DiHETE | Llipoxygenase pathway | 0.8795 |
| *Anaeroglobus geminatus* | PGD2 | Prostaglandins | 0.8653 |
| *Anaeroglobus geminatus* | RvD1 | D-series resolvins | 0.8116 |
| *Anaeroglobus geminatus* | PGF2a | Prostaglandins | 0.8089 |
| *Anaeroglobus geminatus* | PGE2 | Prostaglandins | 0.7940 |
| *Anaeroglobus geminatus* | 12(S)-HHTrE | Prostaglandins | 0.6622 |
| *Actinomyces gerencseriae* | RvE3 | E-series resolvins | 0.8791 |
| *Actinomyces gerencseriae* | LTB4 | Leukotrienes | 0.8771 |
| *Actinomyces gerencseriae* | 5(S)12(S)-DiHETE | Lipoxygenase pathway | 0.8648 |
| *Actinomyces gerencseriae* | PGD2 | Prostaglandins | 0.8514 |
| *Actinomyces gerencseriae* | RvD1 | D-series resolvins | 0.7983 |
| *Actinomyces gerencseriae* | PGF2a | Prostaglandins | 0.7961 |
| *Actinomyces gerencseriae* | PGE2 | Prostaglandins | 0.7815 |
| *Actinomyces gerencseriae* | 12(S)-HHTrE | Prostaglandins | 0.6515 |
| *Capnocytophaga* sp._oral_taxon_336 | 5(S)12(S)-DiHETE | Lipoxygenase pathway | 0.8356 |
| *Capnocytophaga* sp._oral_taxon_336 | LTB4 | Leukotrienes | 0.8024 |
| *Capnocytophaga* sp._oral_taxon_336 | RvE3 | E-series resolvins | 0.7804 |
| *Capnocytophaga* sp._oral_taxon_336 | PGD2 | Prostaglandins | 0.7195 |
| *Capnocytophaga* sp._oral_taxon_336 | RvD1 | D-series resolvins | 0.7191 |
| *Capnocytophaga* sp._oral_taxon_336 | PGF2a | Prostaglandins | 0.6083 |
| *Capnocytophaga* sp._oral_taxon_336 | PGE2 | Prostaglandins | 0.6065 |
| *Capnocytophaga* sp._oral_taxon_336 | Maresin 1 | Maresins | 0.6004 |
| *Selenomonas* sp._oral_taxon_136 | RvE3 | E-series resolvins | 0.9027 |
| *Selenomonas* sp._oral_taxon_136 | LTB4 | Leukotrienes | 0.9026 |
| *Selenomonas* sp._oral_taxon_136 | 5(S)12(S)-DiHETE | Lipoxygenase pathway | 0.8935 |
| *Selenomonas* sp._oral_taxon_136 | PGD2 | Prostaglandins | 0.8714 |
| *Selenomonas* sp._oral_taxon_136 | RvD1 | D-series resolvins | 0.8207 |
| *Selenomonas* sp._oral_taxon_136 | PGF2a | Prostaglandins | 0.8097 |
| *Selenomonas* sp._oral_taxon_136 | PGE2 | Prostaglandins | 0.7956 |
| *Selenomonas* sp._oral_taxon_136 | 12(S)-HHTrE | Prostaglandins | 0.6675 |
| *Selenomonas* sp._oral_taxon_137 | RvE3 | E-series resolvins | 0.8825 |
| *Selenomonas* sp._oral_taxon_137 | LTB4 | Leukotrienes | 0.8811 |
| *Selenomonas* sp._oral_taxon_137 | 5(S)12(S)-DiHETE | Lipoxygenase pathway | 0.8699 |
| *Selenomonas* sp._oral_taxon_137 | PGD2 | Prostaglandins | 0.8537 |
| *Selenomonas* sp._oral_taxon_137 | RvD1 | D-series resolvins | 0.8017 |
| *Selenomonas* sp._oral_taxon_137 | PGF2a | Prostaglandins | 0.7967 |
| *Selenomonas* sp._oral_taxon_137 | PGE2 | Prostaglandins | 0.7823 |
| *Selenomonas* sp._oral_taxon_137 | 12(S)-HHTrE | Prostaglandins | 0.6536 |
| *Selenomonas* sp._oral_taxon_138 | RvE3 | E-series resolvins | 0.9002 |
| *Selenomonas* sp._oral_taxon_138 | LTB4 | Leukotrienes | 0.8991 |
| *Selenomonas* sp._oral_taxon_138 | 5(S)12(S)-DiHETE | Lipoxygenase pathway | 0.8883 |
| *Selenomonas* sp._oral_taxon_138 | PGD2 | Prostaglandins | 0.8704 |
| *Selenomonas* sp._oral_taxon_138 | RvD1 | D-series resolvins | 0.8179 |
| *Selenomonas* sp._oral_taxon_138 | PGF2a | Prostaglandins | 0.8114 |
| *Selenomonas* sp._oral_taxon_138 | PGE2 | Prostaglandins | 0.7969 |
| *Selenomonas* sp._oral_taxon_138 | 12(S)-HHTrE | Prostaglandins | 0.6664 |
| *Selenomonas* sp._oral_taxon_479 | LTB4 | Leukotrienes | 0.8039 |
| *Selenomonas* sp._oral_taxon_479 | 5(S)12(S)-DiHETE | Lipoxygenase pathway | 0.8023 |
| *Selenomonas* sp._oral_taxon_479 | RvE3 | E-series resolvins | 0.8006 |
| *Selenomonas* sp._oral_taxon_479 | PGD2 | Prostaglandins | 0.7675 |
| *Selenomonas* sp._oral_taxon_479 | RvD1 | D-series resolvins | 0.7293 |
| *Selenomonas* sp._oral_taxon_479 | PGF2a | Prostaglandins | 0.7037 |
| *Selenomonas* sp._oral_taxon_479 | PGE2 | Prostaglandins | 0.6928 |
| *Tannerella* sp._oral_taxon_808 | 5(S)12(S)-DiHETE | Lipoxygenase pathway | 0.8774 |
| *Tannerella* sp._oral_taxon_808 | LTB4 | Leukotrienes | 0.8720 |
| *Tannerella* sp._oral_taxon_808 | RvE3 | E-series resolvins | 0.8646 |
| *Tannerella* sp._oral_taxon_808 | PGD2 | Prostaglandins | 0.8230 |
| *Tannerella* sp._oral_taxon_808 | RvD1 | D-series resolvins | 0.7893 |
| *Tannerella* sp._oral_taxon_808 | PGF2a | Prostaglandins | 0.7440 |
| *Tannerella* sp._oral_taxon_808 | PGE2 | Prostaglandins | 0.7341 |
| *Tannerella* sp._oral_taxon_808 | 12(S)-HHTrE | Prostaglandins | 0.6332 |
| *Treponema socranskii* | RvE3 | E-series resolvins | 0.8544 |
| *Treponema socranskii* | LTB4 | Leukotrienes | 0.8537 |
| *Treponema socranskii* | 5(S)12(S)-DiHETE | Lipoxygenase pathway | 0.8441 |
| *Treponema socranskii* | PGD2 | Prostaglandins | 0.8255 |
| *Treponema socranskii* | RvD1 | D-series resolvins | 0.7764 |
| *Treponema socranskii* | PGF2a | Prostaglandins | 0.7686 |
| *Treponema socranskii* | PGE2 | Prostaglandins | 0.7549 |
| *Treponema socranskii* | 12(S)-HHTrE | Prostaglandins | 0.6322 |
