# Supplementary material for: Subgingival Microbiome and Specialized Pro-Resolving Lipid Mediator Pathway Profiles Are Correlated in Periodontal Inflammation
Source: Front Immunol. 2021 Jun 10;12:691216. doi: 10.3389/fimmu.2021.691216 (PMC8222734; doi:10.3389/fimmu.2021.691216)
Supplement: Supplementary file 5 [file Table_4.docx]

**Supplementary Table 4. Mean relative abundance of *Selenomonas* species**

| **Bacterial Species** | **Mean relative abundance (%) in healthy (H)** | **Mean relative abundance (%) in periodontitis before non-surgical therapy (P)** | **Mean relative abundance (%) in periodontitis after non-surgical therapy (A)** |
| --- | --- | --- | --- |
| ***Selenomonas artemidis*** | 0.1920% | 0.0684% | 0.9500% |
| ***Selenomonas dianae*** | 0.0042% | 0.0263% | 0.0120% |
| ***Selenomonas flueggei*** | 0.0049% | 0.0131% | 0.0444% |
| ***Selenomonas noxia*** | 1.0000% | 0.6210% | 2.0000% |
| ***Selenomonas* sp._oral_taxon_126** | 0.0160% | 0.0982% | 0.1140% |
| ***Selenomonas* sp._oral_taxon_134** | 0.0202% | 0.0804% | 0.0611% |
| ***Selenomonas* sp._oral_taxon_136** | 0.0299% | 0.0522% | 0.3230% |
| ***Selenomonas* sp._oral_taxon_137** | 0.0091% | 0.0201% | 0.2650% |
| ***Selenomonas* sp._oral_taxon_138** | 0.0251% | 0.0858% | 0.3980% |
| ***Selenomonas* sp._oral_taxon_146** | 0.0132% | 0.0193% | 0.0688% |
| ***Selenomonas* sp._oral_taxon_388** | 0.0000% | 0.0043% | 0.0000% |
| ***Selenomonas* sp._oral_taxon_479** | 0.0244% | 0.1240% | 0.1540% |
| ***Selenomonas* sp._oral_taxon_919** | 0.0557% | 0.0193% | 0.0342% |
| ***Selenomonas* sp._oral_taxon_936** | 0.0230% | 0.0039% | 0.0000% |
| ***Selenomonas* sp._oral_taxon_937** | 0.0028% | 0.0131% | 0.0013% |
| ***Selenomonas sputigena*** | 0.2050% | 0.5440% | 0.8940% |
| **Sum of relative abundance** | 1.6254% | 1.7933% | 5.3198% |
